# Research on the Hong-Ou-Mandel interference with two independent sources

**DOI:** 10.1038/s41598-019-40720-5

**Published:** 2019-03-07

**Authors:** Si Wang, Chen-Xi Liu, Jian Li, Qin Wang

**Affiliations:** 1 Institute of Quantum Information and Technology, Nanjing University of Posts and Telecommunications, Nanjing, 210003 China; 2 Broadband Wireless Communication and Senser Network Technology, Key Lab of Ministry of Education, NUPT, Nanjing, 210003 China; 3 Telecommunication and Networks, National Engineering Research Center, NUPT, Nanjing, 210003 China

## Abstract

In this paper, we carry out investigation on the HOM interference between two independent photons by using interference filters with different bandwidth both in theory and experiment. Our experimental results are consistent with the theoretical predictions. From the experimental and theoretical results, we find that interference filters with a narrower bandwidth can help to give a larger coherence length, due to the broadening of photon wave-packet in the spatial domain, resulting in an higher interference visibility. Furthermore, a combination of interference filters with different bandwidths may help to achieve a nice balance between coincidence counting rate and interference visibility. Our present work might provide valuable reference for further implementation of HOM interference in the field of quantum information.

## Introduction

Quantum interference plays an important role in quantum information processing, such as quantum cryptography^1^, quantum teleportation^2^, quantum repeater^3^, and linear optical quantum computation^4–6^. The Mach-Zehnder interferometer is usually used to detect the relative phase-shift between the two beams split from a single light source. In 1987, Hong, Ou and Mandel experimentally verified the interference with a beam splitter for two photons from a spontaneous parametric down conversion (SPDC) source, which is known as Hong-Ou-Mandel (HOM) interference. The HOM interference were performed in many experiments, such as the verification of Bell nonlocality^7,8^, quantum key distribution^9^. Furthermore, with the help of polarization beam splitter (PBS), the HOM interferometer can also be performed for quantum logic operation^10^, and multi-photon entangled state generation, such as Greenberger-Horne-Zeilinger (GHZ) state^11,12^, W state^13^, and cluster state^14^. Recently, interference with more photons is used for Boson sampling^15^ and quantum metrology^16^.

For two-photon cases, HOM interference is a second-order coherent effect in quantum optics that is caused by the combination of the indistinguishability and the probability amplitude that both photons are reflected and transmitted by the beam splitter^17^. Both the visibility and the width of HOM dip depend on the degree of indistinguishability of the two photons for temporal, spatial, or spectral character. Up to date, there have been plenty of interesting work studying on HOM interference. For example, Ou and Legero gave early discussions on time-resolved HOM interference between two independent heralded single-photon sources^18,19^; Mosley *et al*. theoretically modelled photon-pairs in factorable states, and then experimentally realized HOM interference without spectral filters^20,21^; Jin *et al*. studied HOM interference between different states, e.g., an pure heralded single-photon state and a weak coherent state^22^, two heralded single-photon sources or two thermal sources^23^; Brańczyk displayed theoretical investigations on spectrally-resolved HOM interferences^24^. Based on previous work, we made further investigation on HOM interference between two heralded photons from two independent SPDC processes, exploring the influence of spectral filtering. On one hand, we theoretically derived the coincidence probability of four-fold HOM interference considering the transmission functions in spectrum of interference filters and simulated it. On the other hand, we experimentally demonstrated the four-fold HOM interference and obtained a number of interference curves with different interference filters. At the bandwidth of 2 nm, both signal and idler from two SPDC sources, the visibility of HOM interference can reach 94.9% ± 2.2% and the full width at half maximum (FWHM) is 254.4 ± 12.4 *μ*m.

This paper is organized as follows. In our paper, we firstly introduce the theory of HOM interference, including a basic model of two-photon interference and four-fold HOM interference with two independent SPDC sources. Secondly, we introduce the basic apparatuses and the experimental process in detail. Finally, the experimental results are discussed and a conclusion is summarized.

## Results

### Theory of HOM Interference

Considering a beam splitter which performs a unitary transformation from two input modes, *a*_1_ and *b*_1_ to two output ones *a*_2_ and *b*_2_,

1 $$\{\begin{array}{rcl}{a}_{2} & = & \frac{1}{\sqrt{2}}({a}_{1}+{b}_{1})\\ {b}_{2} & = & \frac{1}{\sqrt{2}}({a}_{1}-{b}_{1}).\end{array}$$

**Figure 1:**
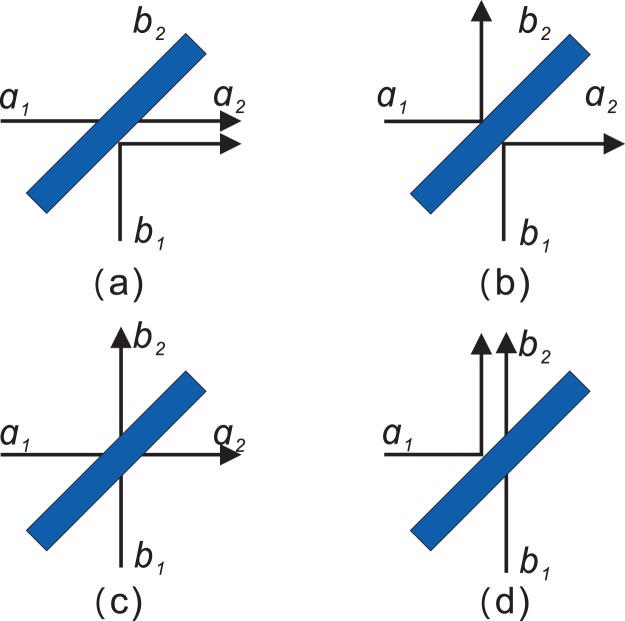
Four different ways for two photons to incident on a beam splitter.

For each input mode, a single photon incidents on the splitter. There are four different input-output relations as shown in Fig. 1.

The input state of two photons before arriving at the beam splitter is

2 $$\begin{array}{l}|{\psi }^{in}\rangle ={a}_{1}^{\dagger }{b}_{1}^{\dagger }|0\rangle ,\end{array}$$

where ${a}_{1}^{\dagger }$ and ${b}_{2}^{\dagger }$ represent creation operators in beam splitter modes, *a* and *b*, respectively. The other properties of photons are labelled by 1 and 2. When some additional properties of two photons are identical, such as polarization, spectral mode^25,26^, temporal mode^27^, arrival time and transverse spatial mode, photons are indistinguishable. As a result, the two photons will always come out from the same output port due the destructive interference between the cases that both photons are transmitting and that both are reflected (see Fig. 1(b,c)). The process of two-photon interference can be modeled with a unitary $\widehat{U}$ as

3 $$\begin{array}{rcl}|{\psi }^{out}\rangle  & = & \widehat{U}|{\psi }^{in}\rangle \\  & = & \widehat{U}({a}_{1}^{\dagger }{b}_{1}^{\dagger })|0\rangle \\  & = & (\frac{1}{\sqrt{2}}{a}_{2}^{\dagger }+\frac{1}{\sqrt{2}}{b}_{2}^{\dagger })(\frac{1}{\sqrt{2}}{a}_{2}^{\dagger }-\frac{1}{\sqrt{2}}{b}_{2}^{\dagger })|0\rangle \\  & = & \frac{1}{2}({a}_{2}^{\dagger }{a}_{2}^{\dagger }-{b}_{2}^{\dagger }{b}_{2}^{\dagger })|0\rangle .\end{array}$$

**Figure 2:**
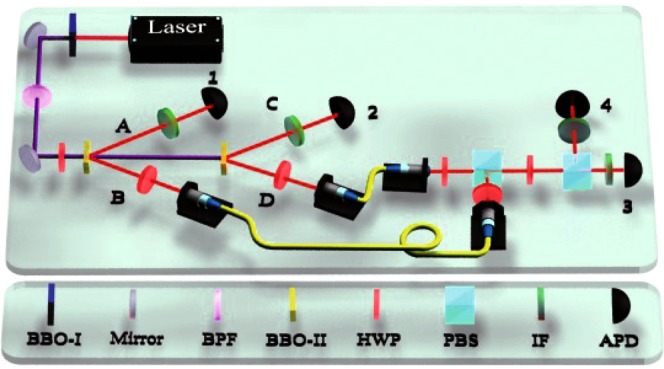
Experimental setup of HOM interference. There are two consecutive spontaneous parametric down-conversion (SPDC) sources and an interferometer. BBO: a *β*-barium borate (BBO) crystal cut for collinear type-I phase-matching; BBO-II: type-II BBO; HWP: half-wave plate; 2 nm IF: interference filter with a full width at half maximum (FWHM) of 2 nm; APD: single-photon detector (The silicon avalanche photodiodes, SPCM-AQRH-13-FC by Excelitas Technologies, are used as single photon detectors, with the typical photon detection efficiency about 63% at 780 nm).

The original HOM interference was performed with two photons from a SPDC source. Here, let’s consider the HOM interference with a polarization beam splitter for two independent photons heralded from two SPDC source. As shown in Fig. 2, the signal and idle photons from a non-collinear type-II beam-like SPDC source are respectively labeled as *A* and *B*. Photon *A* is detected by a single photon detector 1 as a trigger, and the heralded photon *B* is coupled into a single mode fiber and then collimated in horizontal polarization as an input mode of polarization beam splitter (PBS), which is transmittive for horizontally-polarized photon and reflective for vertically-polarized one. In the same way, the photon D, generated by the second SPDC source and heralded by detector 2, is collimated in the vertical polarization and sent to the other input of the PBS. The spatial modes of both photons are aligned carefully to ensure that they come out from the same output port mode, but in orthogonal polarization. An half-wavelength plate is inserted into the mode with the fast axis rotated 22.5 degrees relative to the horizontal direction, which transform the horizontal/vertical polarization into the diagonal/anti-diagonal one. A second PBS separate the horizontal polarization and the vertical one into two different spatial modes, which are coupled by two single-mode fibers, followed by two single-photon detectors 3 and 4. And four interferometric filters are inserted by the detectors to modulate the spectrum of the photons detected.

The joint spectral amplitude function of the signal and idler photon-pair for each SPDC are defined as *f*_1_(*ω*_*A*_, *ω*_*B*_) and *f*_2_(*ω*_*C*_, *ω*_*D*_) respectively, where the joint spectral amplitude is well approximated by *f*(*ω*_*s*_, *ω*_*i*_)∝*α*(*ω*_*s*_, *ω*_*i*_)*φ*(Δ*k*⋅*L*)^28,29^. Here, *α*(*ω*_*s*_, *ω*_*i*_) is the pump spectral envelope function that assumed to be well described by a Gaussian function:

4 $$\begin{array}{rcl}\alpha ({\omega }_{s},{\omega }_{i}) & = & {\rm{\exp }}[-{(\frac{{\omega }_{p}-{\omega }_{{p}_{0}}}{2\pi \sigma })}^{2}]\\  & = & {\rm{\exp }}[-{(\frac{{\omega }_{s}+{\omega }_{i}-{\omega }_{{p}_{0}}}{2\pi \sigma })}^{2}],\end{array}$$

where *ω*_*p*_ and ${\omega }_{{p}_{0}}$ are the frequency and central frequency of the pump light respectively and *σ* denotes the pump spectral bandwidth. The phase-matching function *φ*(Δ*k*⋅*L*) in a nonlinear crystal is given by

5 $$\begin{array}{rcl}\phi ({\rm{\Delta }}k\cdot L) & = & sinc({\rm{\Delta }}k\cdot L/\mathrm{2)}\\  & = & \frac{sin({\rm{\Delta }}k\cdot L/\mathrm{2)}}{{\rm{\Delta }}k\cdot L/2},\end{array}$$

where *L* is the thickness of type-II BBO crystal. Δ*k* can be calculated from the refractive index equation of light and the cutting angle of the crystal. The initial four-photon input state is given by

6 $$\begin{array}{rcl}{|{\psi }^{in}\rangle }_{4} & = & \int \int \int \int {\rm{d}}{\omega }_{A}{\rm{d}}{\omega }_{B}{\rm{d}}{\omega }_{C}{\rm{d}}{\omega }_{D}\\  &  & {a}^{\dagger }({\omega }_{A}){b}^{\dagger }({\omega }_{B}){c}^{\dagger }({\omega }_{C}){d}^{\dagger }({\omega }_{D})\\  &  & {f}_{1}({\omega }_{A},{\omega }_{B}){f}_{2}({\omega }_{C},{\omega }_{D})|0\rangle ,\end{array}$$

where *ω* is the angular frequency. Similar to Eq. (3), the state after the second PBS is then given by

7 $$\begin{array}{ccc}{|{\psi }^{out}\rangle }_{4} & = & \hat{U}{|{\psi }^{in}\rangle }_{4}\\  & = & \frac{1}{2}\int \int \int \int {\rm{d}}{\omega }_{A}{\rm{d}}{\omega }_{B}{\rm{d}}{\omega }_{C}{\rm{d}}{\omega }_{D}{e}^{-i{\omega }_{D}\tau }\\  &  & \,\times \,{f}_{1}({\omega }_{A},{\omega }_{B}){f}_{2}({\omega }_{C},{\omega }_{D}){a}^{\dagger }({\omega }_{A}){c}^{\dagger }({\omega }_{C})\\  &  & \,\times \,[{b}^{\dagger }({\omega }_{B}){b}^{\dagger }({\omega }_{D})+{b}^{\dagger }({\omega }_{D}){d}^{\dagger }({\omega }_{B})\\  &  & \,-\,{b}^{\dagger }({\omega }_{B}){d}^{\dagger }({\omega }_{D})-{d}^{\dagger }({\omega }_{B}){d}^{\dagger }({\omega }_{D})]|0\rangle ,\end{array}$$

where, *τ* is the time delay between path *B* and path *D*. The operator describing a click for detector 1 is written as

8 $$\begin{array}{ccc}{\widehat{P}}_{A} & = & \int {\rm{d}}{\omega }_{1}{a}^{\dagger }({\omega }_{1})|0\rangle \langle \mathrm{0|}a({\omega }_{1}){\varphi }_{1}({\omega }_{1}),\end{array}$$

and similar to other detectors 2, 3, and 4 can be similarly given by

9 $$\begin{array}{ccc}{\widehat{P}}_{B} & = & \int {\rm{d}}{\omega }_{2}{b}^{\dagger }({\omega }_{2})|0\rangle \langle \mathrm{0|}b({\omega }_{2}){\varphi }_{2}({\omega }_{2}),\end{array}$$

10 $$\begin{array}{ccc}{\widehat{P}}_{C} & = & \int {\rm{d}}{\omega }_{3}{c}^{\dagger }({\omega }_{3})|0\rangle \langle \mathrm{0|}c({\omega }_{3}){\varphi }_{3}({\omega }_{3}),\end{array}$$

11 $$\begin{array}{ccc}{\widehat{P}}_{D} & = & \int {\rm{d}}{\omega }_{4}{d}^{\dagger }({\omega }_{4})|0\rangle \langle \mathrm{0|}d({\omega }_{4}){\varphi }_{4}({\omega }_{4}),\end{array}$$

where *ϕ*_1_(*ω*_1_), *ϕ*_2_(*ω*_2_), *ϕ*_3_(*ω*_3_) and *ϕ*_4_(*ω*_4_) are the transmission functions in spectrum of the four interference filters. This transmission function approximate a Gaussian function which can be written as *ϕ*(*ω*) = *exp*{−[(*ω* − *ω*_0_)/2*πσ*_*s*_]^2^}, where *ω*_0_ is the center frequency of single photon and *σ*_*s*_ is a parameter related to the half width of the filter. Then we can calculate the four-fold coincidence probability *P*_4_ between two independent sources by the following formula:

12 $$\begin{array}{rcl}{p}_{4} & = & Tr[{|{\psi }^{out}\rangle }_{4}{\langle {\psi }^{out}|}_{4}{\widehat{P}}_{A}\otimes {\widehat{P}}_{B}\otimes {\widehat{P}}_{C}\otimes {\widehat{P}}_{D}]\\  &  & {\langle {\psi }^{out}|}_{4}{\widehat{P}}_{A}\otimes {\widehat{P}}_{B}\otimes {\widehat{P}}_{C}\otimes {\widehat{P}}_{D}{|{\psi }^{out}\rangle }_{4}.\end{array}$$

Inserting Eqs (7–11) into Eq. (12) and using some operations, e.g. $\langle 0|a(\omega ){a}^{\dagger }({\omega }^{{\rm{^{\prime} }}})|0\rangle =\delta (\omega -{\omega }^{{\rm{^{\prime} }}})$, we can write the coincidence probability as

13 $$\begin{array}{rcl}{p}_{4} & = & \frac{1}{4}\int \int \int \int {\rm{d}}{\omega }_{A}^{^{\prime} }{\rm{d}}{\omega }_{B}^{^{\prime} }{\rm{d}}{\omega }_{C}^{^{\prime} }{\rm{d}}{\omega }_{D}^{^{\prime} }\\  &  & \,\times \int \int \int \int {\rm{d}}{\omega }_{1}{\rm{d}}{\omega }_{2}{\rm{d}}{\omega }_{3}{\rm{d}}{\omega }_{4}\\  &  & \,\times \int \int \int \int {\rm{d}}{\omega }_{A}{\rm{d}}{\omega }_{B}{\rm{d}}{\omega }_{C}{\rm{d}}{\omega }_{D}\\  &  & \,\times {f}_{1}^{\ast }({\omega }_{A}^{^{\prime} },{\omega }_{B}^{^{\prime} }){f}_{2}^{\ast }({\omega }_{C}^{^{\prime} },{\omega }_{D}^{^{\prime} }){f}_{1}({\omega }_{A},{\omega }_{B}){f}_{2}({\omega }_{C},{\omega }_{D})\\  &  & \,\times {\varphi }_{1}({\omega }_{1}){\varphi }_{2}({\omega }_{2}){\varphi }_{3}({\omega }_{3}){\varphi }_{4}({\omega }_{4}){e}^{-i({\omega }_{D}-{\omega ^{\prime} }_{D})\tau }\\  &  & \,\times [\delta ({\omega }_{D}-{\omega }_{2})\delta ({\omega }_{B}-{\omega }_{4})-\delta ({\omega }_{B}-{\omega }_{2})\delta ({\omega }_{D}-{\omega }_{4})]\\  &  & \,\times [\delta ({\omega }_{D}^{^{\prime} }-{\omega }_{2})\delta ({\omega }_{B}^{^{\prime} }-{\omega }_{4})-\delta ({\omega }_{B}^{^{\prime} }-{\omega }_{2})\delta ({\omega }_{D}^{^{\prime} }-{\omega }_{4})]\\  &  & \,\times [\delta ({\omega }_{A}-{\omega }_{1})\delta ({\omega }_{C}-{\omega }_{3})\delta ({\omega }_{A}^{^{\prime} }-{\omega }_{1})\delta ({\omega }_{C}^{^{\prime} }-{\omega }_{3}\mathrm{)].}\end{array}$$

To simplify the Eq. (13), two new functions ${g}_{1}({\omega }_{B},{\omega }_{B}^{^{\prime} })$ and ${g}_{2}({\omega }_{D},{\omega }_{D}^{^{\prime} })$ were defined as

14 $$\begin{array}{l}{g}_{1}({\omega }_{B},{\omega }_{B}^{^{\prime} })=\int {\rm{d}}{\omega }_{A}{f}_{1}^{\ast }({\omega }_{A},{\omega }_{B}){f}_{1}({\omega }_{A},{\omega }_{B}^{^{\prime} }){\varphi }_{1}({\omega }_{A}),\end{array}$$

15 $$\begin{array}{l}{g}_{2}({\omega }_{D},{\omega }_{D}^{^{\prime} })=\int {\rm{d}}{\omega }_{C}{f}_{2}^{\ast }({\omega }_{C},{\omega }_{D}){f}_{2}({\omega }_{C},{\omega }_{D}^{^{\prime} }){\varphi }_{3}({\omega }_{C}\mathrm{).}\end{array}$$

Using the nature of delta functions, therefore the expression of *p*_4_ can be simplified as

16 $$\begin{array}{rcl}{p}_{4} & = & \frac{1}{4}\iint {\rm{d}}{\omega }_{2}{\rm{d}}{\omega }_{4}{\varphi }_{2}({\omega }_{2}){\varphi }_{4}({\omega }_{4})\\  &  & \,\times [{g}_{1}({\omega }_{4},{\omega }_{4}){g}_{2}({\omega }_{2},{\omega }_{2})\\  &  & \,-\,{g}_{1}({\omega }_{4},{\omega }_{2}){g}_{2}({\omega }_{2},{\omega }_{4}){e}^{-i({\omega }_{2}-{\omega }_{4})\tau }\\  &  & \,-\,{g}_{1}({\omega }_{2},{\omega }_{4}){g}_{2}({\omega }_{4},{\omega }_{2}){e}^{-i({\omega }_{4}-{\omega }_{2})\tau }\\  &  & \,+\,{g}_{1}({\omega }_{2},{\omega }_{2}){g}_{2}({\omega }_{4},{\omega }_{4}\mathrm{)].}\end{array}$$

Then we calculated the visibility of the HOM interference by

17 $$\begin{array}{l}V=\frac{{p}_{max}-{p}_{min}}{{p}_{max}},\end{array}$$

where *p*_*max*_ is the four-fold coincidence probability of no interference, and *p*_*min*_ is the four-fold coincidence probability of HOM dip. Based on the derivation above, we known that the coincidence probability which effect the interference visibility is related to the choice of interference filters. In general, *p*_*max*_ = *p*_4_(*τ* = ∞), and *p*_*min*_ = *p*_4_(*τ* = 0).

### Experimental setup

In the experiment, a mode-locked Ti:Sapphire pulsed laser with it’s center wavelength at 780 nm and the pulse length of less than 100 fs, is frequency-doubled into ultraviolet pulses at 390 nm by a *β*-barium borate (BBO) crystal cut for collinear type-I phase-matching. Then the 390 nm laser pumps two *β*-BBOs in sequence. Both of the crystals are 1 mm thick, and cut at angles *θ*_0_ = 42.62° and *ϕ* = 30°, for non-collinear type-II beam-like phase-matching. We get the two photon-pairs in mode *A*&*B* and *C*&*D* respectively. Finally, the photons in mode *A* and mode *C* enter into the single-photon detector respectively through two narrow-band filters with 2 nm FWHM. Two photons in mode *B*&*D* were coupled into a single mode fiber with an aspherical lens (*ModelF*280*FC*−780). What is more, the photons in mode *B* and mode *D* would pass through the interferometer via two optical fibers.

Due to the path difference between the two SPDC sources is about 0.81 m, we use two optical fibers with different lengths to compensate it within 0.5 m, so that these two photons in mode *B*&*D* could enter into the polarization beam splitter (PBS) at the same time. The length that the photons in mode *B* pass through the optical fibers is 2.57 m, *D* pass through the optical fibers is 2.01 m. Due to the fiber refractive index is 1.45, the difference between two optical fibers could approximately compensate the spatial distance between two BBO crystals. In addition, two photons in mode *B*&*D* are collimated and injected into the PBS at the same time by adjusting the distance in mode *D*.

During the experiment, the two-fold coincidence counting rates of both SPDC sources are about 7 kHz. To measure the HOM interference curve from the two independent photons, we record the four-fold coincidence counts while adjusting the position of collimator in mode *D*. The photons from port 3 and port 4 enter into the single-photon detector respectively by two narrow-band filters with different FWHW. The quadruple coincidence counting rate among the single-photon detectors (APDs) 1, 2, 3 & 4 is about 1 Hz when the position is far away from the dip. And, by changing the FWHM of narrow-band filter in port 3 & 4, we can gain many different kinds of HOM interference curves.

## Discussion

It is meaningful to compare a variety of filters that have different kinds of bandwidths. According to the theoretical derivation, the interference visibility is related to the entangled light source and the spectrum of the interference filters. When keeping the spectrum parameter of the down-conversion light source in constant, the interference visibility is only related to the spectra of interference filters. By fixing the narrow-band filters in port 3&4, the spectrum of two triggered photons could be fixed, so the HOM interference curve could be changed by manipulating the interference filters before detectors in port 3 & 4.

**Figure 3:**
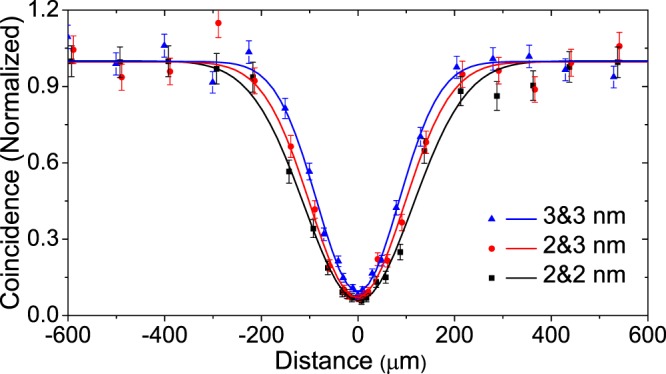
Experimental result of HOM interference curve. The coincidence probability (normalized) for photons from two independent SPDC sources pumped by pulsed lasers. These points represent experimentally measured data, and these full lines represent the results of Gaussian fitting based on these points. In all the interference curves, all interference filters are spectrally centered at 780 nm. The two heralding photons (*A*&*C*) pass through a 2 nm wide (at full width at half maximum) spectral filter. The two heralded photons (*B*&*D*) pass through three different sets of spectral filters: both with a 2 nm filter (square points, V = 94.9% ± 2.2%); *B* with a 2 nm and *D* with a 3 nm filter (circular points, V = 93.0% ± 2.3%); both with a 3 nm filter (Triangle points, V = 90.8% ± 1.8%).

In Fig. 3, we do comparison between HOM interference curves by setting interference filters with different bandwidths (2 nm or 3 nm), where the points are experimental data and curves correspond to the fitting results. Obviously, the interference curve presents a longer coherent length for the sets utilizing interference filters with a narrower bandwidth, see the curve marked with 2 & 2 nm. This is due to the broadening of the photon wave-packet in the spatial domain caused by a narrower interference filter. Meantime, a set of narrower interference filters (2 & 2 nm) can result in a lower coincidence counting rate compared with the set using a broader bandwidth (3 & 3 nm). On the other hand, we find that the HOM interference visibility between two independent sources also varies with the change of filtering. Among the above three sets, the one with two narrower interference filters 2 & 2 nm shows a highest visibility, which is due to the eliminating of the frequency distinguishability of photons by using narrower filters. The set of filters marked with 2 & 3 nm presents medium coincidence counts and a moderate visibility. Therefore, the combination of interference filters with difference bandwidths can give a nice balance between coincidence counting rate and interference visibility among the three curves.

**Figure 4:**
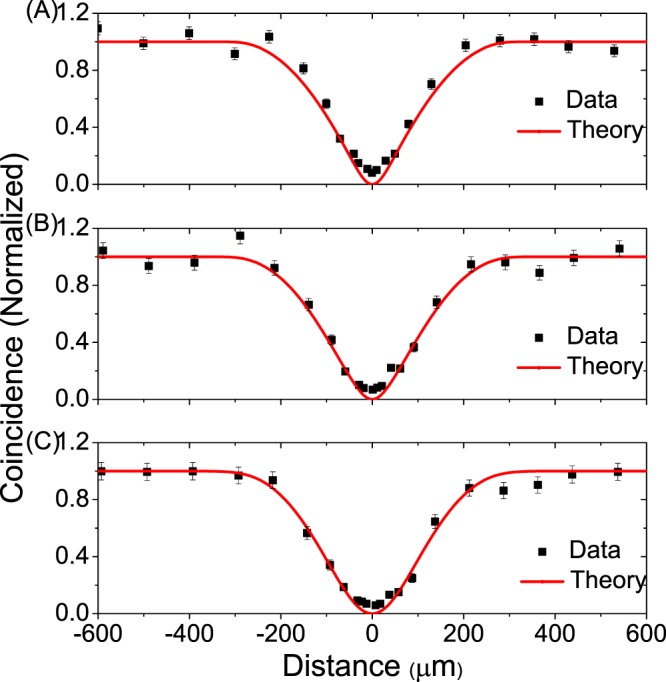
Simulation of HOM interference. The red curve in the figure is the theoretical curve of four-fold coincidence probability in port 1, 2, 3 & 4, and the black point is the actual measured data in the experiment. The error bars show the statistical fluctuation caused by finite data size.

In Fig. 4, we compare the experimental data with our theoretical predictions, where the points represent the experimental data and the curves refer to theoretical calculations. The experimental visibility of interference (VI), theoretical FWHM (TF) and experimental FWHM (EF) are listed out in Table 1, respectively. We find that the theoretical curves and experimental data are roughly in consistent with each other. Since light dispersion and purity of single photons are not taken into consideration in theoretical derivation, the visibility that we obtained is 100%. While in practical experiments, the above issues do exist and thus decrease the interference visibility. That is why the theoretical curve and the experimental data still have a slight deviation, see Fig. 4(A–C).

**Table 1:** The interference filters at output port 3 & 4 are set with: (A) 3 & 3 nm, (B) 2 & 3 nm, (C) 2 & 2 nm; and the interference filters at output port 1 & 2 are all set at 2 nm.

	VI	TF (*μ*m)	EF (*μ*m)
(A)	90.8% ± 1.8%	226.7 ± 0.9	198.9 ± 7.7
(B)	93.0% ± 2.3%	237.5 ± 0.6	226.8 ± 11.5
(C)	94.9% ± 2.2%	248.2 ± 0.2	254.4 ± 12.4

## Conclusion

In summary, we have theoretically and experimentally investigated the four-fold HOM interference curves of two independent SPDC sources by employing different interference filters, getting pretty well consistence between experiment and theory. We find that a narrower interference filter can help to increase the interference visibility of HOM interference. Furthermore, by properly choosing combination of interference filters with different bandwidths, a nice balance between counting rates and interference visibility can be obtained. This work may provide valuable references for the implementation of HOM interferences, and further pave the way towards practical applications of quantum technologies.
